# Holistic rubric vs. analytic rubric for measuring clinical performance levels in medical students

**DOI:** 10.1186/s12909-018-1228-9

**Published:** 2018-06-05

**Authors:** So Jung Yune, Sang Yeoup Lee, Sun Ju Im, Bee Sung Kam, Sun Yong Baek

**Affiliations:** 1 Department of Medical Education, Pusan National University School of Medicine, 49, Busandaehak-ro, Mulgeum-eup, Yangsan-si, Gyeongsangnam-do 50612 Republic of Korea; 2 Family Medicine Clinic and Research Institute of Convergence of Biomedical Science and Technology, Pusan National University Yangsan Hospital, 49, Busandaehak-ro, Mulgeum-eup, Yangsan-si, Gyeongsangnam-do 50612 Republic of Korea

**Keywords:** Clinical assessment, Objective structured clinical examination, Feedback

## Abstract

**Background:**

Task-specific checklists, holistic rubrics, and analytic rubrics are often used for performance assessments. We examined what factors evaluators consider important in holistic scoring of clinical performance assessment, and compared the usefulness of applying holistic and analytic rubrics respectively, and analytic rubrics in addition to task-specific checklists based on traditional standards.

**Methods:**

We compared the usefulness of a holistic rubric versus an analytic rubric in effectively measuring the clinical skill performances of 126 third-year medical students who participated in a clinical performance assessment conducted by Pusan National University School of Medicine. We conducted a questionnaire survey of 37 evaluators who used all three evaluation methods—holistic rubric, analytic rubric, and task-specific checklist—for each student. The relationship between the scores on the three evaluation methods was analyzed using Pearson’s correlation. Inter-rater agreement was analyzed by Kappa index. The effect of holistic and analytic rubric scores on the task-specific checklist score was analyzed using multiple regression analysis.

**Results:**

Evaluators perceived accuracy and proficiency to be major factors in objective structured clinical examinations evaluation, and history taking and physical examination to be major factors in clinical performance examinations evaluation. Holistic rubric scores were highly related to the scores of the task-specific checklist and analytic rubric. Relatively low agreement was found in clinical performance examinations compared to objective structured clinical examinations. Meanwhile, the holistic and analytic rubric scores explained 59.1% of the task-specific checklist score in objective structured clinical examinations and 51.6% in clinical performance examinations.

**Conclusion:**

The results show the usefulness of holistic and analytic rubrics in clinical performance assessment, which can be used in conjunction with task-specific checklists for more efficient evaluation.

## Background

In medical education, a clinical performance assessment (CPA) is a criterion-referenced test that assesses competencies in the care of patients. The main issue is whether the standardization and objectivity of evaluations are reliably maintained in a complex and simulated, clinically relevant, and contextually appropriate setting [1]. In particular, the role of the evaluator as a major factor in the reliability of CPA has often been discussed. Such factors as evaluator expertise, experience, and hawkishness may affect CPA more than the evaluation items, because no single method of setting standards is perfect [2–4].

Holistic rubrics emphasize the use of experts to judge performance assessment. They comprise a comprehensive assessment of the complex multi-faceted characteristics of the tasks undertaken and are based on the overall impression of the experts who implement them. Since performance is not a sum of simple factors, the use of expert holistic rubrics is recognized as a useful evaluation method [5]. However, when assessment times are longer, evaluators generally tend to be more lenient and are more likely to overlook students’ failure factors due to evaluator fatigue [6]. At the beginning of an evaluation, evaluators are reported to exaggerate due to inexperience while, in the latter stage, they exaggerate due to fatigue and comparisons with applicants who have already been assessed. Evaluation of clinical performance through an evaluation matrix has been recommended to avoid evaluator errors [7]. However, it has been pointed out that that to some extent, evaluations using task-specific checklists covering many criteria have difficulties in evaluating competency, and that there is a limit to the effects of the evaluator’s expertise in evaluation [8].

Due to the limitations of task-specific checklist evaluation, it has been proposed that evaluators use a global rating scale [9, 10]. A global rating scale is a holistic rubric that provides a single score based on an overall impression of a student’s clinical performance and can be used in tandem with the task-specific checklist [11]. Global rating scale assessments are easy to develop and use, but there is a high possibility that errors will occur. For example, factors related to applicants may cause a “halo effect”; additionally, the “central tendency,” whereby the evaluator tends to select the middle range, may also cause errors. Holistic rubrics can use behaviorally anchored scales or Likert scales. A global rating scale can be readily used to set standards for performance [12]. However, to use the global rating scale, it is necessary to clearly present pre-shared criteria, i.e., rubrics, to assess learners’ achievements.

In this respect, analytic rubrics have the advantage of reducing evaluator errors on a global rating scale. Analytic rubrics are more reliable than holistic rubrics in that they check the key content, rather than providing a holistic evaluation [13]. Analytic rubrics provide specific feedback according to several sections or dimensions, allow students to identify which factors are missing from the holistic rubric, and enable continuous monitoring [14]. Analytic scoring refers to a process of assigning points to individual traits present in the performance and adding the points to derive single or multiple dimension scores. For example, students with low scores in aseptic manipulation can be educated separately and their progress can be monitored to confirm the degree of improvement in aseptic manipulation ability in the next CPA.

However, little research has been conducted to determine whether holistic rubrics or analytic rubrics are more useful, and to examine how such a rubric-based evaluation approach can be used as a more effective tool than a task-specific checklist evaluation. Therefore, this study examined what factors evaluators consider important in holistic scoring in CPA, and what factors evaluators recognize as useful in holistic grading. The usefulness of these two rubrics was also compared by applying holistic and analytic rubrics in addition to task-specific checklists based on traditional standards. The four overarching research questions guiding the study were as follows: (1) What are the evaluators’ perceptions regarding key factors in determining OSCE and CPX holistic scoring? (2) Are there correlations among the scores of the task-specific checklist, holistic rubric, and analytic rubric in OSCEs and CPXs? (3) Is there agreement between pass and fail frequencies based on the task-specific checklist, holistic rubric, and analytic rubric in OSCEs and CPXs? (4) What is the effect of the holistic rubric and analytic rubric on the task-specific checklist score in OSCEs and CPXs?

## Methods

### Participants and data

This study utilized the data of 126 third-year students who participated in a CPA during two days in 2016, led by Pusan National University School of Medicine. This study was reviewed by the Institutional Review Board of Pusan National University Yangsan Hospital, and given exempt status (IRB No. 05–2017-102). Because we analyzed data retrospectively and anonymously by assigning each subject a distinct number, the institutional review board did not require informed consent from participants. The CPA was operated with 12 stations per day, including six clinical performance examination (CPX) stations and six objective structured clinical examination (OSCE) stations. For each of the CPX and OSCE, 24 professors participated as evaluators with each professor evaluating 31–32 students. The total number of evaluations was 750. Evaluations were used for statistical analysis only if there were data including analytic rubric and global rating scale results, in addition to task-specific checklists as a mandatory assessment method. A total of 292 CPX evaluation cases (38.9%) and 488 OSCE (65.1%) evaluation cases were used as data in the final analysis. In addition, 37 evaluators (77.1%) responded to a questionnaire.

### Materials

A questionnaire was administered to evaluators who participated in the CPA. The most important factor recognized in the global rating scale was as follows: “If you evaluate clinical performance with one item, what factors do you think are most important?. .. In which case, do you think that he/she is a high-performing student? (For example, accuracy, proficiency, sterility, success rate, etc.).” Evaluators assessed whether the holistic rubric for CPA, assigned a score from 0 to 4 and developed according to a score-based criterion, could measure students’ clinical ability to perform. The analytic rubrics were developed based on the results of a questionnaire administered to the faculty focus group. The CPX rubrics allocated 0–3 points each to various fields, including history taking (contents, systematic), physical examination (contents, accuracy), patient education, and attitude. In the case of the OSCE, 0–3 points were allocated for each of the four rubric items of proficiency, accuracy, asepticity, and success. For example, in CPX, we rated students on a 4-point scale from 3 to 0 points in the context of history taking as follows: the student asked the standard patient every single question (3 point), the student missed some questions (2 point), the student missed a lot of questions (1 point), and the student did not do anything (0 point). In the OSCE, we rated students on a 4-point scale from 3 to 0 in asepticity as follows: the whole process was aseptic (3 point), patient safety was ensured but contamination occurred (2 point), contamination threatened patient safety (1 point), and the student did not do anything (0 point). The sum of the analytic rubric items was calculated by weighting the importance of each item. The sum of the CPX analytic rubric was history taking × 40 + physical examination × 30 + patient education × 20 + attitude × 10, while the sum of the OSCE analytic rubric was proficiency × 45 + accuracy × 30 + asepticity × 15 + success × 10. In the case of the task-specific checklist, a score from 0 to 1 or 0 to 2 was allocated for each item, and the sum of the final scores was then obtained. The CPX and OSCE task-specific checklists consisted of 19 to 28 items and 14 to 18 items, respectively.

### Statistical analysis

The contents of the questionnaire responses to important factors in the global rating scale of the CPA were analyzed qualitatively and for frequency. The relationship between the global rating scale, analytic rubric scores, and task-specific checklist total scores was examined using Pearson correlation analysis. Taking into account the task-specific checklist scores, holistic score, and analytic rubric scores, students who were less than 1SD from the average were regarded as having failed the assessment. The pass/fail agreement between the three evaluations was then examined using the Kappa coefficient. Theoretically, if the Kappa value is greater than 0.8, it is a perfect agreement, 0.6 denotes substantial agreement, 0.4 to 0.6 denotes moderate agreement, and 0.2 to 0.4 denotes only a fair agreement [15]. To ascertain which factors had the greatest effect on the task-specific checklist total scores, multiple regression analysis was performed using the holistic rubric score and analytic rubric scores as independent variables and the task-specific checklist total scores as the dependent variable. Statistical analysis of the data was performed using SPSS version 21.0 for Windows (SPSS Inc., Chicago, USA).

## Results

### Evaluators’ perceptions of key factors in determining CPA holistic scoring

In OSCE, accuracy was the most important factor for evaluators who had less than six experiences of evaluation, while evaluators who had participated more than six times recognized proficiency as the most important factor. Evaluators who were professors with less than 10 years of experience recognized accuracy as the most important factor, while professors with more than 10 years of experience considered both accuracy and proficiency to be most important. Overall, evaluators recognized accuracy as the most important factor, followed by proficiency. In CPX, evaluators recognized history taking as the most important factor, followed by physical examination, regardless of the number of evaluation experiences and duration of the working period (Table 1).

**Table 1:** Evaluators’ perceptions of key factors in determining the OSCE and CPX holistic rubric scoring

Factors	OSCE					CPX				
	n	Asepticity	Accuracy	Proficiency	Success	n	History taking	Physical examination	Patient education	Attitude
Evaluation experience										
< 6 times	12	2	7	3	2	16	15	11	3	1
≥6 times	7	1	3	4	–	2	2	–	–	–
Subtotal	19	3	10	7	2	18	17	11	3	1
Faculty career										
< 10 years	7	1	4	1	2	12	9	6	2	–
≥10 years	12	2	6	6	–	6	8	5	1	1
Subtotal	19	3	10	7	2	18	17	11	3	1

Multiple-response

### Relationship among holistic score, analytic rubric scores, and task-specific checklist scores

In the OSCE, the task-specific checklist scores showed a strong positive correlation with holistic score and analytic rubric scores (*r* = 0.751, *P* < 0.001 and *r* = 0.697, P < 0.001, respectively). Holistic score also had a strong positive correlation with analytic rubric scores (*r* = 0.791, *P* < 0.001). In the case of CPX, the task-specific checklist scores showed a strong positive correlation with holistic score and analytic rubric scores (*r* = 0.689, P < 0.001 and *r* = 0.613, P < 0.001, respectively). Holistic score also had a strong positive correlation with analytic rubric scores (*r* = 0.655, *P* < 0.001) (Table 2).

**Table 2:** Correlations among task-specific checklist, holistic rubric, and analytic rubrics scores in the OSCE and CPX

Factor	OSCE (n = 488)				CPX (*n* = 291)			
	Mean ± SD	1	2	3	Mean ± SD	1	2	3
1. Task-specific checklist	12.5 ± 2.9	–			28.1 ± 4.8	–		
2. Holistic rubric	2.4 ± 0.8	0.751*	–		2.4 ± 0.7	0.689*	–	
3. Analytic rubrics	212.0 ± 52.7	0.697*	0.791*	–	400.4 ± 62.1	0.613*	0.655*	–

*P < 0.001, OSCE; objective structured clinical examination, CPX; clinical performance examination

### Inter-rater agreement among holistic score, analytic rubric scores, and task-specific checklist scores

In the OSCE, the task-specific checklist scores showed a moderate agreement with holistic score and analytic rubric scores (Kappa = 0.441, *P* < 0.001 and Kappa = 0.429, P < 0.001, respectively). Holistic score also had a moderate agreement with analytic rubric scores (*r* = 0.512, P < 0.001). Of the students who passed the task-specific checklist, 96.6% passed the holistic rubric and 87.3% passed the analytics rubrics, while of the students who failed the task-specific checklist, 40.0% failed the holistic rubric, and 60% failed the analytic rubrics (Tables 3, 4).

**Table 3:** Pass and fail frequencies based on task-specific checklist, holistic rubric, and analytic rubrics scores in the OSCE and CPX

		Task-specific checklist [*n* (%)]		
		Pass	Fail	Total
OSCE (*n* = 488)				
Holistic rubric	Pass	394 (96.6)	48 (60.0)	442 (90.6)
	Fail	14 (3.4)	32 (40.0)	46 (9.4)
Analytic rubrics	Pass	356 (87.3)	32 (40.0)	388 (79.5)
	Fail	52 (12.7)	48 (60.0)	100 (20.5)
Total		408 (100.0)	80 (100.0)	488 (100.0)
CPX (*n* = 291)				
Holistic rubric	Pass	240 (98.4)	34 (72.3)	274 (94.2)
	Fail	4 (1.6)	13 (27.7)	17 (5.8)
Analytic rubrics	Pass	226 (92.6)	25 (53.2)	251 (86.3)
	Fail	18 (7.4)	22 (46.8)	40 (13.7)
Total		244 (100.0)	47 (100.0)	291 (100.0)

*OSCE*; objective structured clinical examination, CPX; clinical performance examination

Students who were less than 1SD from the average were regarded as having failed the assessment

**Table 4:** Kappa coefficient among task-specific checklist, holistic rubric, and analytic rubrics scores in the OSCE and CPX

	Holistic rubric	Analytic rubrics	Task-specific checklist
OSCE (*n* = 488)			
Holistic rubric	–	0.512*	0.441*
Analytic rubrics	0.512*	–	0.429*
Task-specific checklist	0.441*	0.429*	–
CPX (*n* = 291)			
Holistic rubric	–	0.255*	0.351*
Analytic rubrics	0.255*	–	0.420*
Task-specific checklist	0.351*	0.420*	–

*P < 0.001, *OSCE*; objective structured clinical examination, CPX; clinical performance examination. Students who were less than 1SD from the average were regarded as having failed the assessment. Then, the pass/fail agreement between the three evaluations was examined using the Kappa coefficient

In CPX, the task-specific checklist scores showed a fair agreement with holistic score and analytic rubric scores (Kappa = 0.351, *P* < 0.001 and Kappa = 0.420, P < 0.001, respectively). Holistic score also had a moderate agreement with analytic rubric scores (Kappa = 0.255, P < 0.001). Of the students who passed the task-specific checklist, 98.4% passed the holistic rubric and 92.6% passed the analytic rubrics, while of the students who failed the task-specific checklist, 27.7% failed the holistic rubric, and 46.8% failed the analytic rubrics (Tables 3, 4).

### Explanatory power of holistic rubric and analytic rubrics for task-specific checklist

In the OSCE, multiple regression analyses showed that both holistic score and analytic rubric scores were statistically significant in predicting task-specific checklist scores, with an explanatory power of 59.1% (F = 352.37, *P* < 0.001), while although holistic score was the most influential variable (β =0.534, *P* < 0.001). All variables had variance inflation factors of less than 10 or tolerances of greater than 0.1, which shows that multicollinearity does not exist. In the CPX, multiple regression analyses showed that both holistic score and analytic rubric scores were statistically significant in predicting task-specific checklist scores, with an explanatory power of 51.6% (F = 155.896, P < 0.001), and holistic score (β =0.503, P < 0.001) showed greater explanatory power than analytic rubric scores (β =0.283, P < 0.001) (Table 5).

**Table 5:** Effect of holistic rubric and analytic rubrics on task-specific checklist score by multiple regression in the OSCE and CPX

Independent Variable	B	S.E.	β	t	R	Adj R^2^	F
OSCE (*n* = 488)							
Holistic rubric	1.972	0.346	0.534	11.279*	0.770	0.591	352.37*
Analytic rubrics	0.015	.003	0.274	5.793			
CPX (*n* = 291)0							
Holistic rubric	3.643	0.391	0.503	9.312*	0.721	0.516	155.896*
Analytic rubrics	0.022	0.004	0.283	5.234			

*P < 0.001, OSCE; objective structured clinical examination, CPX; clinical performance examination

## Discussion

Evaluators recognized accuracy as the most important factor in OSCE, and then proficiency. In CPX, history taking was the major factor, followed by physical examination. Based on these results, we developed an analytic rubrics and examined the relationship and agreement among the task-specific checklist, holistic rubric, and analytic rubrics.

In the correlation analysis, both the OCSE and CPX showed a strong positive correlation among holistic score, analytic rubric scores, and task-specific checklist scores. In the Kappa coefficient for the evaluation agreement, the OSCE showed a moderate agreement among task-specific checklist, holistic rubric, and analytic rubrics. In the CPX, however, there was fair agreement between holistic rubric and task-specific checklists or analytic rubrics, and moderate agreement between task-specific checklist and analytic rubrics. Therefore, although task-specific checklists had a strong relationship with holistic rubric and analytic rubrics, there are some discrepancies in the CPX between the three evaluation tools compared to the OSCE. The lower inter-rater agreement in the CPX as compare to OSCE was more influenced by the evaluator, because the evaluation factor of the CPX includes certain subjective items such as attitude or patient education, unlike the OCSE. In addition, in the task-specific checklist evaluation of the CPX, the doctor-patient relationship is evaluated by standardized patients, as opposed to evaluation by a faculty evaluator with clinical experience as a doctor. In a previous study [16], the correlation between the evaluation scores of the faculty evaluator and standardized patient in the physical examination area was 0.91, while the correlation in the doctor-patient relationship was as low as 0.54; this means there may be differences in evaluation areas where objective verification is difficult. It also pointed out that the perception of doctor- patient relationships may not be the same between faculty evaluators and standardized patients. Another previous study [17] on inter-rater agreement between faculty evaluators and standardized patients reported that Kappa values were lower in items related to history taking, but higher in the physical findings, diagnosis, and management items. This evaluation difference between faculty evaluators and standardized patients can be explained in part by the ambiguous scoring criteria of checklist items, lack of training to improve consistency between evaluators, and evaluators’ fatigue [16].

In order to evaluate inter-rater agreement, Kappa coefficient and percent agreement are considered together. In the present study, students who failed the task-specific checklist evaluation often passed the holistic evaluation or the analytical rubrics evaluation in the case of the CPX. These findings mean that it is more difficult for students to pass when evaluated with a large number of evaluation items. However, in the results of this study, it is difficult to determine whether the task-specific checklist, the holistic evaluation, or the analytical rubrics evaluation was more reliable. Previous studies have argued that task-specific checklist evaluation of OSCE cannot evaluate competency and that it is very specific and hierarchical, so it is difficult, using the checklist scores alone to distinguish beginners and experts in terms of problem-solving ability to form accurate hypotheses with minimum information [7, 18]. Therefore, there is a growing emphasis on holistic assessment. Compared to task-specific checklists, holistic grading is superior in reliability and validity, as well as sensitivity to expertized skill level [9, 19], and shows consistently higher internal consistency reliability and higher inter-evaluator reliability [20]. However, further research is needed to generalize our findings to other academic environments.

Regression analysis showed that the holistic rubric and analytic rubrics accounted for 59.1% of the OSCE task-specific checklist score and 51.6% of the CPX task-specific checklist score. The most influential variable in predicting the task-specific checklist score in both the OSCE and CPX was the holistic rubric score. In other words, evaluating a large number of checklist items for CPA may be one way to increase reliability, but a holistic rubric can be a useful tool in terms of efficiency. The evaluator as a clinical physician can quickly assess the degree of clinical performance and know the determinants of overall clinical performance and how well the student is functioning. However, these evaluator determinations cannot be conducted properly by relying on task-specific checklists, and although objective checklists are often used they are not the best way to assess clinical performance. Likewise, specific information on student performance can be difficult to obtain using holistic rubric alone. Therefore, the concurrent use of analytic rubrics evaluation should also be considered for applying evaluation results to real practical situations.

## Conclusion

In summary, this study demonstrates that holistic rubric and analytic rubrics are efficient tools for explaining task-specific checklist scores. Holistic rubric can better explain task-specific checklist scores compared to analytic rubrics. Further validation, however, is required to confirm these findings. Our findings will contribute to the development of evaluation tools to ensure the reliability and efficiency of CPA widely used in medical education, while providing implications for the use of holistic evaluation of professional skills in CPA.

## Abbreviations

CPA: Clinical performance assessment
CPX: Clinical performance examination
OSCE: Objective structured clinical examination
